# The Autoantigenic Proinsulin B-Chain Peptide B11-23 Synergises with the 70 kDa Heat Shock Protein DnaK in Macrophage Stimulation

**DOI:** 10.1155/2018/4834673

**Published:** 2018-12-09

**Authors:** Elias Blasius, Elke Gülden, Hubert Kolb, Christiane Habich, Volker Burkart

**Affiliations:** ^1^Institute for Clinical Diabetology, German Diabetes Center, Leibniz Center for Diabetes Research, D-40225 Düsseldorf, Germany; ^2^West German Center of Diabetes and Health, Düsseldorf Catholic Hospital Group, D-40591 Düsseldorf, Germany; ^3^German Center for Diabetes Research (DZD), D-85764 München-Neuherberg, Germany

## Abstract

**Background:**

Heat shock proteins (Hsp) act as intracellular chaperones and in addition are used as adjuvant in vaccines of peptides complexed with recombinant Hsp. By interacting with autologous peptides, Hsp may promote the induction of autoimmune reactivity.

**Objective:**

Here, we analysed whether the effect of Hsp on macrophages is modulated by insulin peptides known to interact with Hsp.

**Results:**

Combinations of the 70 kDa Hsp DnaK with peptide B11-23 from the core region of the proinsulin B-chain induced the release of the inflammatory mediators interleukin-6, tumor necrosis factor *α*, and interleukin-1*β* from cells of human and murine macrophage lines. In parallel, there was high-affinity binding of B11-23 to DnaK. DnaK mixed with peptides from other regions of the insulin molecule did not stimulate cytokine secretion. DnaK alone induced little cytokine production, and peptides alone induced none.

**Conclusion:**

The macrophage-stimulating potential of Hsp70 family proteins when combined with the proinsulin B-chain peptide B11-23 may contribute to the immunodominance of this peptide in the development of beta cell-directed autoimmunity in type 1 diabetes.

## 1. Introduction

Type 1 diabetes (T1D) results from the immune-mediated destruction of insulin-producing pancreatic beta cells leading to insulin deficiency in affected individuals [1]. The hallmark of the pathogenesis of T1D is the development of autoimmune reactivity against beta cell constituents. Insulin is a dominant beta cell-associated autoantigen [2, 3]. Within the insulin molecule, the strongest autoimmune T cell reactivity occurs towards the B-chain region B9-25 [1, 3].

Innate immune cells, such as macrophages and dendritic cells, are critical in the induction and progression of beta cell-damaging immunologic processes, because of their antigen-presenting properties [4, 5]. Studies on the characterization of endogenous signals controlling macrophage activity identified members of the heat shock protein (Hsp) family as mediators with pronounced macrophage-stimulating potential [6].

Under physiological conditions, Hsp serve as intracellular chaperones which transiently bind (poly-)peptides to assist their biosynthesis and transport [7]. However, Hsp released from stressed or dying cells can act as potent endogenous danger signals preferentially targeting cells of the innate immune system [8, 9]. Moreover, extracellular Hsp are able to confer increased immunogenicity to their chaperoned peptides by improving their recognition by antigen-presenting, innate immune cells [10, 11]. This property enables Hsp to contribute to the activation of antigen-specific host defence mechanisms but may also promote the induction of immune reactivity against autologous peptides [12].

We therefore used a model system to analyse the macrophage-stimulating potential of insulin peptides and heat shock protein DnaK. The bacterial 70 kDa chaperone DnaK is a well-characterised analogue of mammalian Hsp70 chaperones, of which peptide-binding properties have been analysed in detail [13]. Macrophage stimulation was assessed by determining the release of representative mediators relevant in the immunopathogenesis of T1D, particularly tumor necrosis factor *α* (TNF*α*) and interleukin-1*β* (IL-1*β*), identified as potent beta cell-damaging cytokines [14], as well as interleukin-6 (IL-6), playing an important role as immunostimulatory cytokine in diabetes [15, 16].

## 2. Materials and Methods

### 2.1. Monocyte/Macrophage Cell Lines

The human monocyte line MonoMac 6 (DSMZ, Braunschweig, Germany) was cultivated in RPMI 1640 medium (PAA Laboratories, Linz, Austria) containing an oxaloacetate pyruvate insulin (OPI) supplement (Sigma-Aldrich Chemie GmbH, Steinheim, Germany), 2 mM L-glutamine, antibiotics (120 mg/l penicillin, 200 mg/l streptomycin), and 10% FCS (Life Technologies, Eggenstein, Germany), at 37°C and 5% CO_2_. The mouse macrophage line J774A.1 (ATCC, Manassas, VA, USA) was cultivated in RPMI 1640 medium supplemented as mentioned above, plus 1 mM sodium pyruvate, but without the OPI supplement [8].

### 2.2. Stimulation of Macrophages for Cytokine Production

For stimulation of cytokine production, J774A.1 or MonoMac 6 cells were seeded in 96-well, flat-bottom plates (2 × 10^5^ cells/well). The cells remained untreated (medium control) or were exposed to 10 ng/ml lipopolysaccharide (LPS) (*Escherichia coli* O26:B6, Sigma-Aldrich Chemie GmbH) or to various concentrations of recombinant DnaK (Stressgen, Victoria, BC, Canada), 13mer peptides derived from the proinsulin molecule [17] (IHB, Leiden University Medical Center, Peptide Synthesis Facility, Leiden, The Netherlands) (Table 1), or to mixtures of DnaK and peptides. DnaK and peptides were analysed by the Limulus amoebocyte lysate assay (Lonza AG, Basel, Switzerland) for endotoxin contamination. LPS was not detectable. The full-length DnaK amino acid sequence (638 amino acids) was derived from the *E. coli* paradigm strain K-12 (Uni-Prot ID: P0A6Y8).

### 2.3. Quantification of Cytokine Production by Macrophages

LPS-, DnaK-, peptide-, or DnaK/peptide-induced cytokine production by macrophages was determined in the culture supernatants. Based on previous work showing different kinetics of the release of macrophage mediators after exposure to Hsp (and LPS) [8, 18, 19], TNF*α* levels were measured after 6 h and IL-6 and IL-1*β* levels were measured after 24 h of incubation in the absence or presence of the reagents. Cytokine concentrations were quantified by the use of specific ELISAs (OptEIA Set, BD Pharmingen) or multiplex technology according to the manufacturer's instructions [20, 21].

### 2.4. Competition of Complex Formation between DnaK and Reduced Carboxymethylated Lactalbumin (RCMLA) by Insulin Peptides

For competition binding assays, 70 nM DnaK was incubated with 40 *μ*M RCMLA (Sigma-Aldrich Chemie GmbH) in buffer containing 25 mM Tris-HCl, 20 mM HEPES, 47.5 mM KCl, and 2.25 mM Mg(OAc)_2_ in the absence or presence of increasing concentrations of competing peptides (2 h, 37°C) [22]. DnaK-RCMLA complexes were separated from free DnaK and DnaK-peptide complexes by electrophoresis on native polyacrylamide gels (6%). After immunoblotting, the DnaK-RCMLA complexes were visualised by sequential application of a mouse anti-DnaK antibody 8E2/2 (Stressgen), a rabbit anti-mouse IgG-HRP antibody (Stressgen), and an ECL kit (Pierce Biotechnology/Thermo Fisher Scientific Inc., Rockford, IL, USA) in a Lumi-Imager Workstation (Roche Applied Science, Mannheim, Germany) [22]. All experiments were repeated at least three times.

### 2.5. Statistical Analyses

Data were expressed as mean ± SD. Statistical analyses were performed using ANOVA with Bonferroni post-hoc analysis. Differences were considered statistically significant with *P* < 0.05. All statistical analyses were performed using the Prism software package version 5 (GraphPad Software, San Diego, CA, USA).

## 3. Results

### 3.1. Induction of Proinflammatory Macrophage Mediators by Combinations of DnaK and the Insulin Peptide B11-23

**Figure 1:**
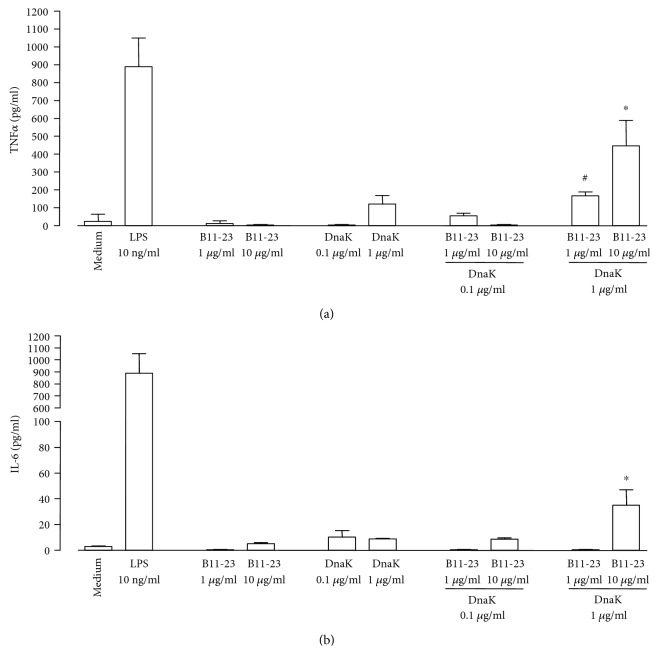
Induction of inflammatory mediators from murine macrophages by the insulin B-chain peptide B11-23 and DnaK. J774A.1 cells were cultivated in the absence (medium) or presence of LPS, peptide B11-23, DnaK, or peptide-DnaK combinations at the indicated concentrations. Levels of TNF*α* (a) and IL-6 (b) in the culture supernatants were determined by ELISA. Data show mean + SD from 3 experiments performed in triplicates. ^∗^*P* < 0.05 compared to medium control, peptide, or DnaK alone. ^#^*P* < 0.05 compared to medium control.

Combinations of DnaK and the insulin B-chain-derived peptide B11-23 stimulated the release of the proinflammatory mediators TNF*α* and IL-6 from cells of the murine macrophage line J774A.1 (Figure 1). A mixture of 1 *μ*g/ml DnaK and 10 *μ*g/ml of the peptide induced the accumulation of 446 ± 143 pg/ml TNF*α* after 6 h (Figure 1(a)) and of 35 ± 12 pg/ml IL-6 after 24 h (Figure 1(b)). Exposure of the macrophages to DnaK alone (1 *μ*g/ml) resulted in only a small increase of IL-6 levels above background (8.7 ± 0.5 pg/ml, *P* < 0.01). In all other cases, TNF*α* or IL-6 production was in the range of the medium control after coincubation of macrophages with either DnaK or peptide B11-23 alone. Bacterial LPS (10 ng/ml) was used as a positive control and induced the accumulation of high amounts of TNF*α* (889 ± 228 pg/ml after 6 h, Figure 1(a)) and of IL-6 (1086 ± 149 pg/ml after 24 h, Figure 1(b)).

**Figure 2:**
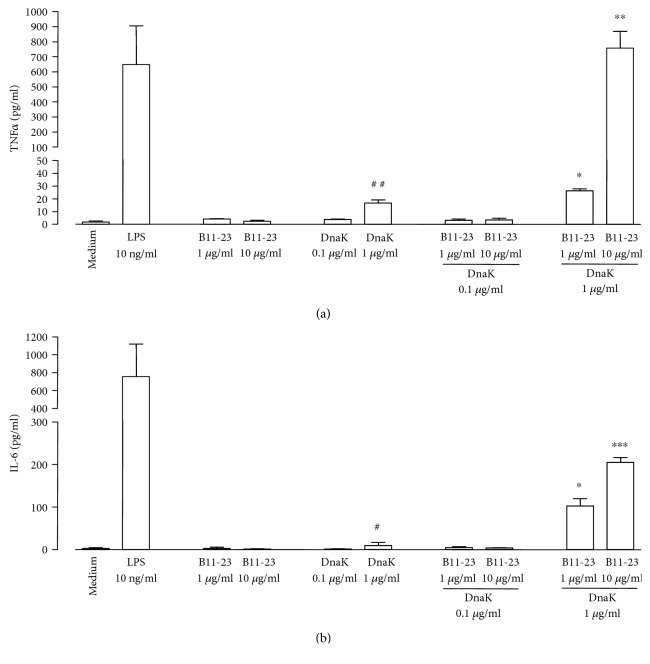
Induction of inflammatory mediators from human monocytes by the insulin B-chain peptide B11-23 and DnaK. MonoMac 6 cells were cultivated in the absence (medium) or presence of LPS, peptide B11-23, DnaK, or peptide-DnaK combinations at the indicated concentrations. Levels of TNF*α* (a) and IL-6 (b) in the culture supernatants were determined by ELISA. Data show means + SD from 3 experiments performed in triplicates. ^∗^*P* < 0.05, ^∗∗^*P* < 0.01, and ^∗∗∗^*P* < 0.001 compared to medium control, peptide, or DnaK alone. ^#^*P* < 0.05 and ^##^*P* < 0.01 compared to medium control.

Similar observations were made with cells of the human monocyte line MonoMac 6 (Figure 2). A combination of DnaK (1 *μ*g/ml) and the peptide B11-23 (10 *μ*g/ml) induced the release of 757 ± 110 pg/ml TNF*α* after 6 h (Figure 2(a)) and 572 ± 42 pg/ml IL-6 after 24 h (Figure 2(b)). Again, DnaK or the peptide alone did not induce significant amounts of cytokines, except for low levels of TNF*α* (16.7 ± 2.5 pg/ml, *P* < 0.005) and IL-6 (9.8 ± 7.0 pg/ml, *P* < 0.05) in response to the higher concentration of DnaK. Incubation of macrophages with LPS had a strong stimulatory effect (649 ± 257 pg/ml TNF*α* after 6 h; 757 ± 365 pg/ml IL-6 after 24 h).

### 3.2. Interaction of the Insulin B-Chain Peptide B11-23 with DnaK

**Figure 3:**
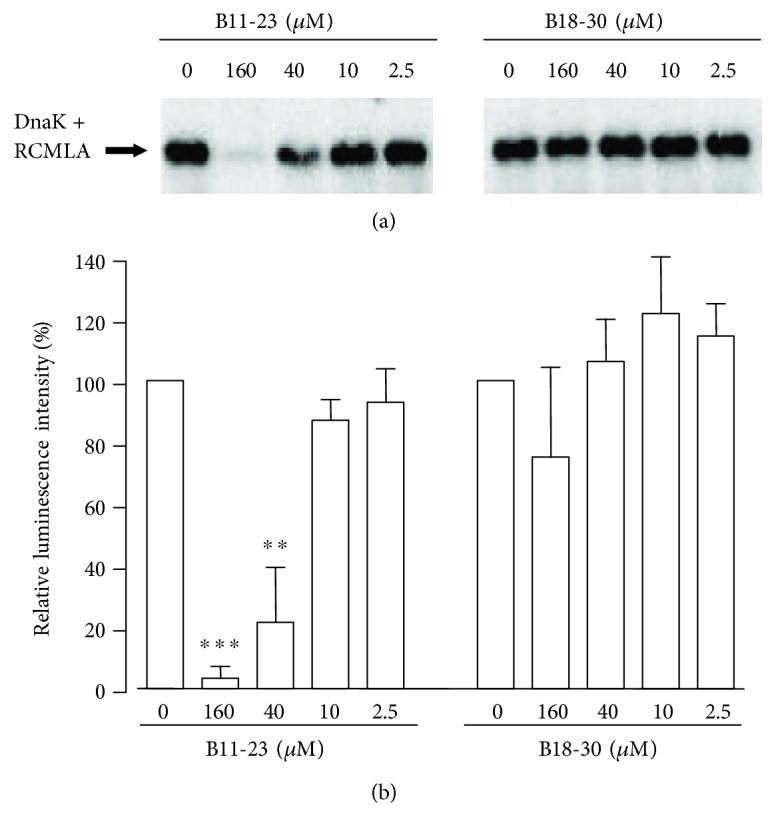
Competition of complex formation between DnaK and RCMLA by insulin B-chain peptides. DnaK was incubated with RCMLA in the absence or presence of increasing concentrations of the peptides B11-23 or B18-30. DnaK-RCMLA complexes were separated from free DnaK and DnaK-peptide complexes by native PAGE and visualised after blotting by the use of anti-DnaK antibodies and ECL. (a) Result of a representative blot. (b) The strengths of the resulting signals were quantified by a luminescence detection system and are shown as relative luminescence intensity setting the signals of DnaK-RCMLA samples in the absence of competing peptide as 100%. Data show means + SD from 3 separate experiments. ^∗∗^*P* < 0.01 and ^∗∗∗^*P* < 0.001 compared to the luminescence intensity of the DnaK-RCMLA sample in the absence of peptide. Peptides at 2.5 *μ*M correspond to 3.7 *μ*g/ml of peptides, 10 *μ*M correspond to 14.6 *μ*g/ml of peptides, and 40 *μ*M correspond to 58.6 *μ*g/ml of peptides.

The ability of insulin peptides to interact with DnaK was analysed in a competition binding assay which allows to assess the DnaK-binding capacity of a peptide/protein by determining its ability to compete with binding of the high-affinity DnaK ligand RCMLA to the chaperone [22]. As indicated by the dose-dependent reduction of the signal strength of the DnaK-RCMLA complexes (Figure 3(a), left), the 13mer peptide B11-23 efficiently interfered with the interaction between the chaperone and its ligand. At a concentration of 160 *μ*M, the peptide almost completely prevented DnaK-RCMLA complex formation (Figure 3(b)). The effect of the peptide was reduced in a dose-dependent manner and was almost lost at a concentration of 2.5 *μ*M. To exclude unspecific interference of peptides with DnaK-RCMLA interaction, we analysed the effect of the peptide B18-30 encompassing 13 amino acids of the C-terminal region of the proinsulin B-chain, partially overlapping with the sequence of peptide B11-23 (Table 1). In contrast to peptide B11-23, the peptide B18-30, even at a high concentration of 160 *μ*M, did not interfere with DnaK-RCMLA complex formation (Figure 3(a), right) as demonstrated by the failure of the peptide to displace RCMLA from the chaperone (Figure 3(b)).

### 3.3. Comparative Analyses of the Macrophage Stimulatory Capacity of the Peptides B11-23 and B18-30 in Combination with DnaK

**Figure 4:**
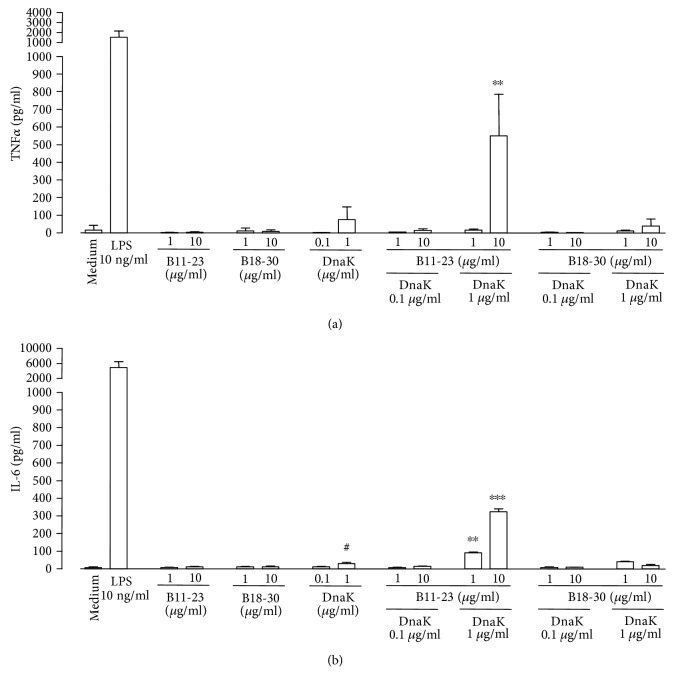
Comparison of the macrophage stimulatory capacity of the insulin B-chain peptides B11-23 and B18-30 in combination with DnaK. J774A.1 cells were incubated in the absence (Medium) or presence of LPS, peptides B11-23 or B18-30, DnaK, or peptide-DnaK combinations at the indicated concentrations. Levels of TNF*α* (a) and IL-6 (b) in the culture supernatants were determined by ELISA. Data show means + SD from 3 experiments performed in triplicates. ^∗∗^*P* < 0.01 and ^∗∗∗^*P* < 0.001 compared to medium control, peptides, or DnaK alone. ^#^*P* < 0.05 compared to medium control.

In comparative analyses, cells of the J774A.1 line were exposed to mixtures of DnaK and the peptides B11-23 or B18-30. Combinations of DnaK (1 *μ*g/ml) and peptide B11-23 (10 *μ*g/ml) induced the release of 549 ± 236 pg/ml TNF*α* after 6 h (Figure 4(a)) or 322 ± 17 pg/ml IL-6 after 24 h (Figure 4(b)), whereas combinations of the chaperone with the peptide B18-30 had no effects. Again, the peptides alone exhibited no stimulatory activities. DnaK alone did not induce significant secretion of TNF*α* but low levels of IL-6, as also shown in Figure 1. Exposure of the cells to LPS (10 ng/ml) induced the release of high amounts of TNF*α* (1527 ± 596 pg/ml after 6 h, Figure 4(a)) and IL-6 (4918 ± 1548 pg/ml after 24 h, Figure 4(b)).

### 3.4. Macrophage Stimulatory Effects of Peptides Derived from Proinsulin A- and B-Chains or the C-Peptide

Further studies were performed with two peptides (B18-30, C8-20) lacking recognisable binding capacity for DnaK and two peptides (A8-20, C19-31) with moderate binding activity for DnaK [17]. The peptides did not elicit a significant cytokine response when added to macrophages of the human cell line MonoMac 6. Also, all peptides did not induce significant cytokine secretion when mixed with DnaK (Table 2). By contrast, the stimulatory activity of the mixture of peptide B11-23 with DnaK was reproduced and also seen for IL-1*β* (Table 2).

## 4. Discussion

We report here on a synergism of the Hsp70 analogue DnaK and the proinsulin B-chain peptide B11-23 in activating proinflammatory cytokine production in macrophages. The studies described here were performed with DnaK since it is the best characterised member of the hsp70 family. Because of the chaperone function of hsp70, there is only limited room for variations in binding specificity between different species, as documented by the remarkable conservation of amino acid sequences between bacteria, mice, and men [23]. This is further illustrated by comparing the full-length amino acid similarity between *E. coli* and man (74.1%) with the higher similarity of the amino acid sequence of the substrate-binding domain in both species (86.1%). The similarity of the substrate-binding domain between mice and man even is 99.1% [24–26]. The B11-23 sequence includes a potent binding motif of DnaK [13], a hydrophobic core of four to five amino acids with a dominant role of leucine (LYLV in the case of peptide B11-23) often flanked by regions enriched in basic residues (only one region with E in the case of peptide B11-23). The general features of substrate binding are conserved in the hsp70 family [27].

Hsp alone, such as that occurring in the extracellular compartment, can act as “danger antigen” and stimulate macrophage function, and this property is independent of their chaperone activity [6, 8]. In the experiments described here, low concentrations of DnaK were chosen in order to meet the range of Hsp70 serum levels of healthy persons [28]. In this concentration range, DnaK induced no or minimal cytokine production from the murine and the human macrophage/monocyte lines J774A.1 and MonoMac 6, in contrast to high cytokine levels induced by LPS. Both cell lines had been established in previous studies to investigate macrophage reactivity towards inflammatory signals [29]. The use of monocyte/macrophage lines allowed to work with standardised and reproducible assay conditions. In order to avoid results that are not representative but are features of a unique cell line, we included two different monocyte/macrophage lines from two quite distant species, mouse and man.

The size of peptides used in the current experiments was chosen to fit with the peptide-binding cleft of chaperones of the hsp70 family [23]. Peptides of this size usually are not able to activate macrophages or other innate immune cells. Indeed, none of the five peptides was found to induce cytokine secretion from macrophages, even when added at a concentration of 10 *μ*g/ml. However, stimulatory effects were observed when the proinsulin peptide B11-23 and DnaK were added together to the macrophage lines. Mixtures of the peptide and DnaK induced the release of TNF*α* and IL-1*β*, identified as beta cell-damaging mediators [14] as well as of IL-6, an immunostimulatory cytokine [15, 16]. The finding that DnaK/B11-23 mixtures stimulate cells of both murine and human macrophage lines implies that the responsiveness to this distinct proinsulin peptide/Hsp combination represents a general functional property of macrophages.

We hypothesized that the synergism between peptide B11-23 and DnaK results from an association of the insulin peptide with the chaperone. Assessing a possible interaction between DnaK and the peptide B11-23 by the use of a competition binding assay with RCMLA as high-affinity ligand [22] in fact revealed displacement of the ligand from DnaK by peptide B11-23, but not by peptide B18-30 derived from the C-terminal region of the proinsulin B-chain. These findings are supported by the results of our previous studies on the interaction between DnaK and insulin B-chain peptides [17]. Subsequent comparative analyses of the macrophage-activating potential of both peptides confirmed the synergism between peptide B11-23 and DnaK but revealed the incapability of peptide B18-30 to raise a macrophage response in the presence of the chaperone.

One other insulin peptide with little binding to DnaK, C8-20, and two peptides with moderate binding to DnaK, A8-20 and C19-31 [17], did not synergise with DnaK to induce cytokine secretion. This suggests that peptide B11-23 is unique in this regard, possibly because of its high-affinity binding to DnaK.

At high concentrations, as they may occur in inflamed tissue, extracellular Hsp are potent activators of macrophages or dendritic cells and this adjuvant-like property is being used for tumor vaccines consisting of recombinant Hsp plus isolated tumor cell peptides [11, 30]. We find here that at low, nonstimulatory concentrations of DnaK, admixture of a high-affinity peptide B11-23 results in a potent macrophage stimulatory complex.

Whether this observation is related to the finding that the peptide B11-23 is a dominant target of pancreatic islet autoreactivity in animal and human T1D remains to be determined. We compared B11-23 with peptides representing secondary autoantigenic epitopes of the insulin molecule, peptides A8-20 and C19-31 [31–33]. Despite their moderate-affinity binding to DnaK [17], synergism in the induction of cytokine secretion was not observed. It is conceivable that other strong DnaK-binding peptides may also stimulate macrophage activity. For efficient antigen presentation, such peptides probably must be present at local high concentration and must exhibit appropriate binding characteristics for major histocompatibility antigens.

## 5. Conclusions

Taken together, our results demonstrate that the insulin peptide B11-23 potentiates the ability of the 70 kDa chaperone DnaK to stimulate proinflammatory macrophage activity. Our results may be relevant in view of the increased expression of Hsp 70 in islets exposed to inflammatory conditions and in patients with T1D [34, 35].

## Figures and Tables

**Table 1:** Amino acid sequences of peptides.

Proinsulin peptide		AA sequence
Origin	Peptide	
A-chain	A8-20	*TSICSLYQLENYC*
B-chain	B11-23	*LVEALYLVCGERG*
B-chain	B18-30	*VCGERGFFYTPKT*
C-peptide	C8-20	*GQVELGGGPGAGS*
C-peptide	C19-31	*GSLQPLALEGSLQ*

Amino acid sequences of selected 13mer peptides derived from proinsulin (A- or B-chain or C-peptide) [13, 17].

**Table 2:** Release of IL-6, TNF*α*, and IL-1*β* from MonoMac 6 cells exposed to proinsulin-derived 13mer peptides alone or in combination with DnaK.

	IL-6 (ng/ml)^a^	TNF*α* (ng/ml)^a^	IL-1*β* (ng/ml)^a^
Medium	<0.1	<0.1	<0.2
LPS	76.3 ± 9.0b^b^	45.44 ± 8.14^b^	12.70 ± 1.02^b^
B11-23	<0.1	<0.1	<0.2
B11-23 + DnaK	19.0 ± 4.3^c^	10.9 ± 1.9^d^	5.5 ± 1.0^d^
B18-30	<0.1	<0.1	<0.2
B18-30 + DnaK	<0.1	<0.1	<0.2
A8-20	<0.1	<0.1	<0.2
A8-20 + DnaK	5.7 ± 4.9	0.6 ± 0.9	<0.2
C8-20	<0.1	<0.1	<0.2
C8-20 + DnaK	7.7 ± 5.5	<0.1	<0.2
C19-31	<0.1	<0.1	<0.2
C19-31 + DnaK	<0.1	<0.1	<0.2

MonoMac 6 cells were incubated in the absence (medium) or in the presence of LPS (10 ng/ml) or DnaK (1 *μ*g/ml) in the absence or presence of proinsulin peptides (10 *μ*g/ml). Concentrations of TNF*α* were determined after 6 h of incubation and concentrations of IL-6 and IL-1*β* were determined after 24 h of incubation in the culture supernatants. Results from peptide-DnaK combinations were corrected by cytokine levels induced by stimulation with DnaK alone. ^a^Data are given as means ± SD from three experiments performed in triplicates. The multiplex system used here for cytokine measurements yielded lower results than ELISA (Figure 2), but the relative differences between LPS, peptide B11-23, and peptide B11-23 + DnaK were not affected. ^b^*P* < 0.001 for the difference to medium. ^c^*P* < 0.01 for the differences between peptide and peptide + DnaK. ^d^*P* < 0.001 for the differences between peptide and peptide + DnaK.

## Data Availability

The data used to support the findings of this study are available from the corresponding author upon request.

## References

[1] Eisenbarth G. S., Moriyama H., Robles D. T. (2002). Insulin autoimmunity: prediction/precipitation/prevention type 1A diabetes. *Autoimmunity Reviews*.

[2] Jasinski J. M., Eisenbarth G. S. (2005). Insulin as a primary autoantigen for type 1A diabetes. *Clinical and Developmental Immunology*.

[3] Nakayama M., Babaya N., Miao D. (2006). Long-term prevention of diabetes and marked suppression of insulin autoantibodies and insulitis in mice lacking native insulin B9-23 sequence. *Annals of the New York Academy of Sciences*.

[4] Zipris D. (2008). Innate immunity and its role in type 1 diabetes. *Current Opinion in Endocrinology, Diabetes and Obesity*.

[5] Kolb H., von Herrath M. (2017). Immunotherapy for type 1 diabetes: why do current protocols not halt the underlying disease process?. *Cell Metabolism*.

[6] Wallin R. P. A., Lundqvist A., Moré S. H., von Bonin A., Kiessling R., Ljunggren H.-G. (2002). Heat-shock proteins as activators of the innate immune system. *Trends in Immunology*.

[7] Deuerling E., Bukau B. (2004). Chaperone-assisted folding of newly synthesized proteins in the cytosol. *Critical Reviews in Biochemistry and Molecular Biology*.

[8] Chen W., Syldath U., Bellmann K., Burkart V., Kolb H. (1999). Human 60-kDa heat-shock protein: a danger signal to the innate immune system. *The Journal of Immunology*.

[9] Habich C., Burkart V. (2007). Heat shock protein 60: regulatory role on innate immune cells. *Cellular and Molecular Life Sciences*.

[10] Tamura Y., Yoneda A., Takei N., Sawada K. (2016). Spatiotemporal regulation of Hsp90–ligand complex leads to immune activation. *Frontiers in Immunology*.

[11] Binder R. J., Srivastava P. K. (2005). Peptides chaperoned by heat-shock proteins are a necessary and sufficient source of antigen in the cross-priming of CD8^+^ T cells. *Nature Immunology*.

[12] Eggleton P. (2003). Stress protein-polypeptide complexes acting as autoimmune triggers. *Clinical and Experimental Immunology*.

[13] Rüdiger S., Germeroth L., Schneider-Mergener J., Bukau B. (1997). Substrate specificity of the DnaK chaperone determined by screening cellulose-bound peptide libraries. *The EMBO Journal*.

[14] Cnop M., Welsh N., Jonas J. C., Jorns A., Lenzen S., Eizirik D. L. (2005). Mechanisms of pancreatic *β*-cell death in type 1 and type 2 diabetes: many differences, few similarities. *Diabetes*.

[15] Hunter C. A., Jones S. A. (2015). IL-6 as a keystone cytokine in health and disease. *Nature Immunology*.

[16] Kristiansen O. P., Mandrup-Poulsen T. (2005). Interleukin-6 and diabetes: the good, the bad, or the indifferent?. *Diabetes*.

[17] Burkart V., Siegenthaler R. K., Blasius E. (2010). High affinity binding of hydrophobic and autoantigenic regions of proinsulin to the 70 kDa chaperone DnaK. *BMC Biochemistry*.

[18] Wollenberg G. K., DeForge L., Bolgos G., Remick D. G. (1993). Differential expression of tumor necrosis factor and interleukin-6 by peritoneal macrophages in vivo and in culture. *American Journal of Pathology*.

[19] Burkart V., Kim Y. E., Hartmann B. (2002). Cholera toxin B pretreatment of macrophages and monocytes diminishes their proinflammatory responsiveness to lipopolysaccharide. *The Journal of Immunology*.

[20] Märker T., Kriebel J., Wohlrab U., Burkart V., Habich C. (2014). Adipocytes from New Zealand obese mice exhibit aberrant proinflammatory reactivity to the stress signal heat shock protein 60. *Journal of Diabetes Research*.

[21] Marker T., Sell H., Zillessen P. (2012). Heat shock protein 60 as a mediator of adipose tissue inflammation and insulin resistance. *Diabetes*.

[22] Vandenbroeck K., Alloza I., Brehmer D. (2002). The conserved helix C region in the superfamily of interferon-*γ*/interleukin-10-related cytokines corresponds to a high-affinity binding site for the HSP70 chaperone DnaK. *Journal of Biological Chemistry*.

[23] Radons J. (2016). The human HSP70 family of chaperones: where do we stand?. *Cell Stress and Chaperones*.

[24] Rauch J. N., Zuiderweg E. R. P., Gestwicki J. E. (2016). Non-canonical interactions between heat shock cognate protein 70 (Hsc70) and Bcl2-associated anthanogene (BAG) co-chaperones are important for client release. *Journal of Biological Chemistry*.

[25] Takayama S., Bimston D. N., Matsuzawa S. (1997). BAG‐1 modulates the chaperone activity of Hsp70/Hsc70. *The EMBO Journal*.

[26] Mayer M. P. (2013). Hsp70 chaperone dynamics and molecular mechanism. *Trends in Biochemical Sciences*.

[27] Rüdiger S., Buchberger A., Bukau B. (1997). Interaction of Hsp70 chaperones with substrates. *Nature Structural & Molecular Biology*.

[28] Wright B. H., Corton J. M., el-Nahas A. M., Wood R. F. M., Pockley A. G. (2000). Elevated levels of circulating heat shock protein 70 (Hsp70) in peripheral and renal vascular disease. *Heart and Vessels*.

[29] Wright E. L., Quenelle D. C., Suling W. J., Barrow W. W. (1996). Use of mono mac 6 human monocytic cell line and J774 murine macrophage cell line in parallel antimycobacterial drug studies. *Antimicrobial Agents and Chemotherapy*.

[30] Shevtsov M., Multhoff G. (2016). Heat shock protein–peptide and HSP-based immunotherapies for the treatment of cancer. *Frontiers in Immunology*.

[31] Vaughan K., Peters B., Mallone R., von Herrath M., Roep B. O., Sette A. (2013). Navigating diabetes-related immune epitope data: re-sources and tools provided by the Immune Epitope Da-tabase (IEDB). *Immunome Research*.

[32] Mallone R., Brezar V., Boitard C. (2011). T cell recognition of autoantigens in human type 1 diabetes: clinical perspectives. *Clinical and Developmental Immunology*.

[33] Pathiraja V., Kuehlich J. P., Campbell P. D. (2015). Proinsulin-specific, HLA-DQ8, and HLA-DQ8-transdimer–restricted CD4^+^ T cells infiltrate islets in type 1 diabetes. *Diabetes*.

[34] Eizirik D. L., Welsh M., Strandell E., Welsh N., Sandler S. (1990). Interleukin-1*β* depletes insulin messenger ribonucleic acid and increases the heat shock protein hsp70 in mouse pancreatic islets without impairing the glucose metabolism. *Endocrinology*.

[35] Oglesbee M. J., Herdman A. V., Passmore G. G., Hoffman W. H. (2005). Diabetic ketoacidosis increases extracellular levels of the major inducible 70-kDa heat shock protein. *Clinical Biochemistry*.

